# First report of the prevalence of Shiga toxinproducing *Escherichia coli* in ground beef in Quindío, Colombia

**DOI:** 10.7705/biomedica.7004

**Published:** 2023-12-01

**Authors:** Valentina Giraldo-Rubio, Brayan Stiven Arango-Gil, Claudia Viviana Granobles-Velandia

**Affiliations:** 1 Grupo de Inmunología Molecular (GYMOL), Centro de Investigaciones Biomédicas, Universidad del Quindío, Armenia, Colombia Universidad del Quindío Centro de Investigaciones Biomédicas Universidad del Quindío Armenia Colombia

**Keywords:** Shiga-toxigenic *Escherichia coli* (STEC), Shiga toxin, ground beef, butchery, prevalence., *Escherichia coli* Shiga-toxigénica (STEC), carne molida, carnicería, prevalencia

## Abstract

**Introduction.:**

Shiga toxin-producing *Escherichia coli* (STEC) is a foodborne pathogen associated with clinical cases of diarrhea in humans. Its main virulence factors are the Shiga toxins (Stx1 and Stx2). Cattle are the main reservoir of STEC, and many outbreaks in humans have been related to the consumption of undercooked ground beef contaminated with this pathogen.

**Objective.:**

To determine the prevalence of STEC in ground beef commercialized in all the butcher shops of a township in the department of Quindío and to characterize the virulence genes of the strains found.

**Materials and methods.:**

Thirty ground beef samples were taken in three different times; *stx* genes and other STEC virulence factors *(eae, ehxA*, *saa*) were detected by multiplex PCR.

**Results.:**

The overall prevalence of STEC was 33.33 % (10/30 positive samples). We isolated eight non-O157 (LEE-negative) strains with four different genetic profiles: *stx*_2_ / *stx*_2_-*ehxA*-*saa* / *stx*_1_-*stx*_2_-*ehxA*-*saa / stx*_1_-*saa*.

**Conclusion.:**

This is the first report on the prevalence of STEC in ground beef in a township in the department of Quindío.

## Resumen

**Introducción.:**

*Escherichia coli* productora de toxina Shiga (STEC) es un agente patógeno de origen alimentario asociado a casos clínicos de diarrea en humanos; sus principales factores de virulencia son las toxinas Shiga (Stx1 y Stx2). El principal reservorio de STEC es el ganado bovino y muchos brotes en humanos se han relacionado con el consumo de carne mal cocida contaminada con este agente patógeno.

**Objetivo.:**

El objetivo de este trabajo fue determinar la prevalencia de STEC en carne molida comercializada en todas las carnicerías de un municipio del departamento del Quindío y caracterizar los genes de virulencia de las cepas encontradas.

**Materiales y métodos.:**

Se tomaron 30 muestras de carne molida en tres momentos diferentes; se detectaron los genes *stx* y otros factores de virulencia de STEC (*eae*, *ehxA*, *saa*) mediante PCR Multiplex.

**Resultados.:**

Los resultados mostraron una prevalencia global de STEC del 33,33 % (10/30 muestras positivas). En total se aislaron ocho cepas STEC no-O157 (LEE-negativas) con cuatro perfiles genéticos diferentes: *stx*_2_ / *stx*_2_-*ehxA*-*saa* / *stx*_1_-*stx*_2_-*ehxA*-*saa / stx*_1_-*saa*.

**Conclusión.:**

Este es el primer reporte que muestra la prevalencia de STEC en carne molida en un municipio del departamento del Quindío.

Shiga toxin-producing *Escherichia coli* (STEC) is an important foodborne pathogen, involved in several outbreaks of diarrhea in humans worldwide. STEC infections can cause gastroenteritis, hemorrhagic colitis, and hemolytic uremic syndrome in severe cases ^1^. Shiga toxins (Stx1 and Stx2) encoded by temperate bacteriophages are the main virulence factor, responsible for the pathological characteristics and severe complications of STEC infection ^2^. Stx2 is up to 1.000 times more cytotoxic than Stx1 and strains producing Stx2 are frequently associated with the development of the hemolytic uremic syndrome ^3^. Likewise, other virulence genes that encode adhesion proteins as well as enterohemolysin (*ehxA*) contribute to the pathogenicity of STEC ^2^.

Among STEC, serogroup O157 has been recognized as one of the major foodborne pathogens due to its ability to cause severe infections in humans ^4^. Recently, other non-O157 serotypes belonging to six O serogroups (O26, O111, O103, O121, O45, and O145) referred to as the “big six” have been recognized as serotypes of public health importance due to their increasing association with human disease ^5^^,^^6^. A common characteristic in these serotypes is the presence of the pathogenicity island locus of enterocyte effacement, where a type III secretion system (T3SS) is encoded, which allows the bacteria to adhere to the epithelium and cause alterations in the architecture and physiology of colonic epithelial cells ^7^.

On the other hand, a new subset of STEC strains that do not carry the locus of enterocyte effacement (LEE) pathogenicity island (LEE-negative) has emerged in recent years. These strains have been isolated from cases of severe disease in countries such as Australia ^8^ and Argentina ^9^. In the absence of locus of enterocyte effacement, the molecular mechanism of adhesion to the epithelium is unknown. However, several virulence factors such as adhesins and toxins encoded mainly in pathogenicity islands have been found; the locus of aggregation and agglutination ^10^, the subtilase encoding pathogenicity island ^11^ and the locus of proteolysis activity ^12^ are some of them.

Cattle are considered the main reservoir of STEC, the transmission to humans can occur through the consumption of contaminated meat products from cattle, such as hamburgers and ground beef ^13^. Contamination of meat usually occurs during slaughter by contact with feces or by crosscontamination during handling in the butcher shops ^14^. Likewise, ground beef is particularly a product of concern for STEC contamination because during the grinding operation, liquids are released, which facilitates the movement of bacteria and the exposure of a greater surface where STEC can colonize ^15^. In Colombia, prevalent data for STEC is unknown due to the lake of surveillance in the food chain for this specific pathogen. This study aimed to determine the prevalence of STEC in ground beef commercialized in all the butcher shops of a township in the department of Quindío and to characterize the virulence genes of the isolated strains.

## Materials and methods

### Sampling

We obtained 30 ground beef samples from all butcher shops (10 shops) from a township located in Quindío department, Colombia with approximately 28.000 inhabitants. Samples were taken biweekly in each butchery three times from March to May 2019. Samples were packed in sterile bags, stored on ice, transported to the Biomedical Research Center (CIBM) of the *University of Quindío* and processed immediately. This study was descriptive cross-sectional research carried out for convenience.

### Sample processing

Ground beef samples were enriched as previously described ^16^ with some modifications. Briefly, 65 g of ground beef were homogenized with 250 ml of buffered peptone water and shaken at 37 °C for 18 h. An aliquot of enriched peptone water was streaked on MacConkey agar and incubated for 24 h at 37 °C. After incubation, a part of the confluent growth was inoculated into 15 mL of brain-heart infusion (BHI) broth and cultured for 4 h at 37 °C with shaking. Then, an aliquot was boiled after diluting it in sterile doubledistilled water and used as a DNA template for PCR.

### Detection of stx genes

Multiplex PCR was used to detect *stx* genes (*stx*_*1*_ and *stx*_*2*_ ) according to the method proposed by Paton and Paton ^17^. Briefly, the conditions for the PCR were 35 cycles of 95 °C for 1 minute, and 65 °C for 2 min in the first 10 cycles, decreasing to 60 °C by cycle 15, and finally, 72 °C for 1.5 min increasing to 2.5 min from cycles 25 to 35. Taq DNA Polymerase (Invitrogen) was used and the PCR products were analyzed by electrophoresis in 2% agarose gels. Positive samples for *stx*_*1*_ , *stx*_*2*_ or both, were stored at -80 °C. They were then processed to isolate and characterize STEC strains genotypically. Samples were labeled according to the sampling number (S1 to S3), and each butchery was assigned a letter (A to J). The O157 STEC strain ATCC 43888 was used as a positive control for the *eae* gene, and the 103 STEC strain ^18^ was used as a positive control for genes *stx*_*1*_ , *stx*_*2*_ , *saa* and *ehxA*. As a negative control, we used *E. coli* strain ATCC 25922 which does not possess STEC virulence genes.

### STEC isolation, virulence profiling and molecular serogrouping

Positive samples for *stx* genes were cultured in three types of selective agars; MacConkey agar, eosin methylene blue agar and trypticase soy agar modified with novobiocin and incubated for 24 h at 37 °C. Between 50 to 250 individual colonies were examined for the presence of *stx* genes. Positive colonies for the Shiga toxin genes were analyzed by multiplex PCR for the presence of *stx*_*1*_ , *stx*_*2*_ , intimin protein encoded by *eae* gene (marker of LEEpositive strains), the auto-agglutinating adhesin encoded by *saa* gene (marker of LEE-negative strains) and the enterohemolysin encoded by *ehxA* gene ^17^^,^^19^. Each isolated STEC strain was confirmed as *E. coli* by detecting the gene encoding the universal stress protein (*usp*A) by PCR ^20^. The isolated STEC strains were screened through specific primers for the presence of the following serotypes: O157 (F-CAGGTGAAGGTGGAATGGTTGTC, R-TTAGAATTGAGACCATCCAATAAG), O45 (F-GGGCTGTCCAGACAGTTCAT, R-TGTACTGCACCAATGCACCT) and O26 (F-AGGGTGCGAATGCCATATT, R-GACATAATGACATACCACGAGCA) ^21^.

### Data analysis

To calculate overall prevalence, the total number of positive samples during the three samplings was divided by 30, which represents the total number of samples taken. Likewise, we calculated a sampling prevalence by taking the number of positive samples and dividing them by the total of sampled butcher shops.




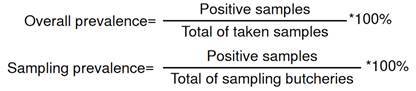




## Results

### STEC prevalence

In this study, a total of 30 ground beef samples were obtained from all the butcheries of a township. An overall prevalence of 33.33 % was found and the sampling prevalence was 40 % in sampling one, 20 % in sampling two and 40 % in sampling three (table 1). In the analyzed butcheries, we found at least one positive sample in six shops. Butchery D showed the highest frequency with three positive samplings (3/3), followed by butchery C and F, with two positive samplings (2/3), and finally butchery A, B and J with one positive sampling (1/3). In the butchery (G, H, I) there were no positive samples for *stx* genes in the three samplings (0/3).

**Table 1:** Frequency of positive samples for stx genes in the sampled butcheries

Butchery^a^	Sampling one	Sampling two	Sampling three	Frequency
A	-	-	+	1/3
B	-	+	-	1/3
C	+	-	+	2/3
D	+	+	+	3/3
E	-	-	-	0/3
F	+	-	+	2/3
G	-	-	-	0/3
H	-	-	-	0/3
I	-	-	-	0/3
J	+	-	-	1/3
Sampling prevalence	40%	20%	40%	Total:10/30 (33.33 %)

^a^ The letter (A to J) corresponds to the butchery sampled**.**

### Characterization of STEC isolates

Different selective culture media were used for the isolation of STEC strains. However, we could isolate and characterize eight strains from ten STEC positive samples. All isolates were PCR positive for the *uspA* gene, indicating that they are *E. coli.* Multiplex PCR showed that 4/8 (50 %) carried only the *stx*_2_ gene, 1/8 (12.5 %) only possessed the *stx*_1_ gene and 3/8 (37.5 %) carried both *stx*_1_ and *stx*_2_ genes. Regarding the other virulence genes, *saa* and *exh*A were detected in 6/8 (75 %) and 5/8 (62.5 %) of the isolates, respectively. None of the eight isolates carried the *eae* gene (table 2). Four different genetic profiles were observed: *stx*_2_ / *stx*_2_-*exhA*-*saa* / *stx*_1_-*stx*_2_-*exhA*- *saa / stx*_1_-*saa*. The use of different media allowed the successful isolation of the eight STEC strains. Finally, none of the eight isolates were PCR positive for serogroups O157, O26 and O45.

**Table 2:** Genetic virulence profile of STEC strains isolated in each butchery

Strains_a_	stx_1_	stx_2_	saa	exhA	eae	O157	O45	O26	UspA
S1-BC	-	+	+	+	-	-	-	-	+
S1-BD	-	+	+	+	-	-	-	-	+
S1-BF	+	+	+	+	-	-	-	-	+
S2-BB	-	+	-	-	-	-	-	-	+
S3-BA	+	+	+	+	-	-	-	-	+
S3-BC	+	-	+	-	-	-	-	-	+
S3-BD	+	+	+	+	-	-	-	-	+
S3-BF -	-	+	-	-	-	-	-	-	+

^a^ Strains were labeled according to the sampling number (S1 to S3), the letter (A to F) corresponds to the sampled butchery.

## Discussion

Shiga toxin-producing *Escherichia coli* (STEC) is a pathotype of *E. coli* characterized by its ability to produce potent cytotoxins of the Shiga toxin family (Stx). Cattle is the main reservoir of STEC and its transmission occurs principally by the consumption of contaminated meat products. Ground beef is especially susceptible to bacterial contamination because of its greater surface where STEC can colonize. In this study, we isolated and determined the virulence factors of STEC strains and showed the prevalence of STEC in ground beef from all the butcheries of a township in the Quindío department, Colombia.

We found an overall prevalence of 33.33 % (10/30 positive samples) of STEC in ground beef samples; we could isolate STEC strains in 80 % (8/10) of the positive samples. This is the first report of STEC prevalence in ground beef in the Quindío department, and the first study that sampled all the butcheries of a township in Colombia.

Ground beef has been subject to STEC detection in Colombia; in Pamplona, Colombia, 78 % of positive samples for *stx* genes were found in raw meat, and a higher percentage of positive samples (64 %) were found in ground beef. They could only isolate STEC strains in 13 % of the positive samples being all of them non-O157 ^22^. Other studies in Montería and Bogotá showed 10 % and 6.06 % of positive samples for STEC in ground beef respectively, although both studies focused on searching for O157:H7 strains, only the first one could detect that serotype ^23^^,^^24^.

At least one positive sample was found in 6/10 butcher shops and we observed differences in sampling prevalence. The recurrent presence of STEC in some butcheries could be caused by different reasons; it has been shown that slaughterhouses are important sources of STEC transmission into the food chain because during slaughter, intestinal contents or feces may be in contact with the meat, or cross-contamination during processing of it. Different studies have found a positive correlation in STEC prevalence between bovine feces and bovine carcasses, demonstrating that cross contamination can occur in slaughterhouses ^25^^-^^27^. In the butcheries, STEC could be spread by cross-contamination during handling either by the contact of contaminated food with utensils; surfaces and equipment without disinfection after use, or by storing meat at inadequate temperatures ^25^.

Differences in virulence factors were observed in STEC isolates, 87.5 % carried the *stx*_2_ gene alone or in combination with *stx*_1_. It has been shown that Stx2 is 1.000 times more toxic than Stx1 and the probability of developing hemolytic uremic syndrome in infections by strains harboring *stx*_2_ is higher ^3^^,^^28^. Although Stx production is considered essential, other virulence factors contribute to its pathogenicity, such as the presence of adhesive proteins. In our study, 75 % of the strains carried the *saa* gene and 62.5 % the *exhA* gene. *Saa* is an adhesin identified mainly in LEE-negative strains ^29^ that contribute to intestinal colonization and the pathogenicity of STEC strains ^30^. On the other hand, *exhA* is a cytolysin, produced by both LEEpositive and LEE-negative STEC strains, frequently detected in strains associated with hemolytic uremic syndrome and plays an important role in the pathogenicity by lysing erythrocytes and releasing hemoglobin as a potential source of iron for bacteria ^31^.

It is important to highlight those two isolates (25 %) tested negative for the *saa* gene and none of the eight STEC strains tested positive for the *eae* gene. According to the literature, a Hes protein member of the Hra family recently identified and described in LEE-negative STEC strains confers an adherent phenotype to *E. coli* HB101 strain (non-adherent) in epithelial cells, this adhesin (encoded in LAA pathogenicity island) could be important for the pathogenesis of LEE-negative strains ^10^^,^^32^. Likewise, other adhesins, such as F18 and EibG, rarely studied, have been described and could be present in these isolates ^33^^,^^34^.

All isolated STEC strains were shown to be non-O157; our results showed that they tested negative for serotypes O157, O26 and O45. In recent years, six non-O157 serotypes known as “the big six” have been recognized as a growing public health concern ^35^. In the USA, non-O157 STEC serotypes are the leading cause of acute diarrhea over O157 strains, their incidence increased from 0.19 per 100,000 in 2007 to 0.79 per 100,000 in 2014 ^13^. Currently, at least 158 serogroups of *E. coli* carrying *stx* genes are known ^36^, and 129 O-serogroups have been associated with clinical cases of diarrhea in humans in sporadic infections and outbreaks ^6^.

In South America, STEC infections appear to be more common in the southernmost countries of the continent, where STEC surveillance is mandatory because STEC infections are a significant public health issue. In contrast, the magnitude of the problem is still unknown in other South American countries, including Colombia ^37^^,^^38^. A major presence of non-O157 strains has been evidenced in Colombia ^18^^,^^22^^-^^24^^,^^39^. Likewise, Calle *et al.* 2019 ^40^ conducted a study in five slaughterhouses that supply 50 % of the bovine cattle consumed in the country. They found serotype O45 was the most represented, followed by O121, O103 and O26. However, some serotypes were not identified, the serotype O157 was identified only in 4.8 % of the samples demonstrating the low prevalence of this serotype observed in other studies ^23^^,^^41^.

In this way, we demonstrate the presence of STEC non-O157 in ground beef in butcher shops of a township and identify the virulence factors of the isolated strains.

Given the recent importance of non-O157 serotypes (LEE-negative) and that in Colombia they seem to be the predominant serotypes, a greater effort should be made to surveillance STEC in the supply food chain and to identify the serotypes present in the country as well as their associated virulence factors. It should be clarified that cooking meat at temperatures higher than 70 °C achieves destruction of the bacteria and does not represent a risk to the consumer; however, since there is no surveillance of STEC-associated diseases in humans, there is no prevalence data that would allow us to know the current situation of this pathogen in Colombia.
